# Amyloid accumulation is a late event in sporadic Alzheimer's disease-like pathology in nontransgenic rats

**DOI:** 10.18632/oncotarget.2751

**Published:** 2014-12-24

**Authors:** Natalia A. Stefanova, Natalia A. Muraleva, Elena E. Korbolina, Elena Kiseleva, Kseniya Yi. Maksimova, Nataliya G. Kolosova

**Affiliations:** ^1^ Institute of Cytology and Genetics, Novosibirsk, Russia; ^2^ Institute of Mitoengineering, Moscow, Russia; ^3^ Siberian State Medical University, Tomsk, Russia; ^4^ Novosibirsk State University, Novosibirsk, Russia

**Keywords:** Alzheimer's disease, amyloid β, tau, synaptic losses, neurodegeneration, mitochondria, OXYS rats

## Abstract

The amyloid cascade hypothesis posits that deposition of the amyloid β (Aβ) peptide in the brain is a key event in the initiation of Alzheimer's disease (AD). Nonetheless, it now seems increasingly unlikely that amyloid toxicity is the cause of sporadic AD, which leads to cognitive decline. Here, using accelerated-senescence nontransgenic OXYS rats, we confirmed that aggregation of Aβ is a later event in AD-like pathology. We showed that an age-dependent increase in the levels of Aβ_1–42_ and extracellular Aβ deposits in the brain of OXYS rats occur later than do synaptic losses, neuronal cell death, mitochondrial structural abnormalities, and hyperphosphorylation of the tau protein. We identified the variants of the genes that are strongly associated with the risk of either late-onset or early-onset AD, including *App, Apoe4, Bace1, Psen1, Psen2, and Picalm*. We found that in OXYS rats nonsynonymous SNPs were located only in the genes *Casp3* and *Sorl1*. Thus, we present proof that OXYS rats may be a model of sporadic AD. It is possible that multiple age-associated pathological processes may precede the toxic amyloid accumulation, which in turn triggers the final stage of the sporadic form of AD and becomes a hallmark event of the disease.

## INTRODUCTION

Alzheimer's disease (AD) is the most common type of age-related dementia worldwide, with dramatically increasing incidence as a consequence of ageing of the population. According to the *amyloid hypothesis*, which dominated the research of AD pathogenesis for more than 20 years, accumulation of soluble amyloid β (Aβ) into toxic oligomers and amyloid plaques initiates a pathogenic cascade leading to accumulation of the hyperphosphorylated tau protein in neurofibrillary tangles, to mitochondrial dysfunction, loss of synapses, neuronal cell death, and, ultimately, a loss of cognitive function [1–3]. The evidence supporting this hypothesis includes genetic effects of the dominantly inherited familial early-onset form of AD, which accounts for ~5% of all cases, involving mutations in amyloid precursor protein (*APP*), presenilin 1 (*PSEN1*), or *PSEN2* genes [4]. The factors that initiate (or affect the risk and onset) of Aβ accumulation in sporadic late-onset AD, which accounts for ~95% of all AD cases, remain poorly understood [5]. A growing body of evidence indicates that amyloid toxicity (resulting from a gain-of-function mutation) is unlikely to be the cause of sporadic AD [2]. Alternative explanations are that sporadic AD dementia is not the result of a single cause but rather of multiple age-associated processes that erode brain structure and function gradually, making it vulnerable to degeneration; combined with the conditions that trigger the events of AD, the above processes result in accelerated neuronal and synaptic losses and in cognitive decline [2].

A major problem in AD research is the lack of an animal model that accurately replicates the human disease. It seems that rodents have fewer propensities to aggregate Aβ than the human one. This shortage makes it difficult to study the underlying mechanisms and to explore additional risk factors and therapeutic approaches to AD. Recently, we showed that accelerated-senescence OXYS rats are a promising model for studies of the mechanisms of neurodegenerative processes similar to those seen in AD [6–8]. The behavioral alterations and learning and memory deficits develop by age 3 months, i.e., simultaneously with first signs of neurodegeneration. With age, neurodegenerative changes in OXYS rats become amplified, accompanied by overproduction of AβPP, accumulation of Aβ, and hyperphosphorylation of tau. Nonetheless, it remains unknown what comes first during the erosion of brain structure and function, and what mechanisms might be behind the primary neuronal dysfunction.

Here we characterized nontransgenic OXYS rats as a model of sporadic AD and report that OXYS rats exhibit age-related accumulation of soluble Aβ and phosphorylation of the insoluble tau protein, as well as synaptic losses, neuronal cell death, and mitochondrial structural abnormalities. Also, we determined variants of the genes that are associated with AD and could contribute to AD-like pathology in OXYS rats.

## RESULTS

### Amyloid β deposits and increased amounts of Aβ in OXYS rats

**Figure 1:**
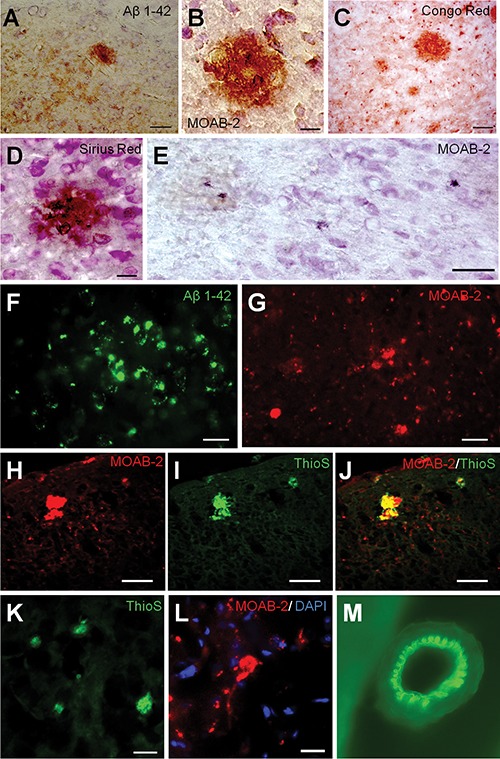
Deposition of Aβ variants in the brain of OXYS rats **(A, B, E–H)**, and **(J)** Brain slices were immunostained for Aβ, with an Aβ_1–42_- and MOAB-2-specific antibody, respectively. Panel **(C)** shows the Kongo Red-stained plaques in the cortex, and the same plaques were identified by counterstaining with Sirius Red **(D)**. Photomicrographs **(I, J)** and **(K)** demonstrate that the fibrillar Aβ deposits can be detected by Thioflavin-S. Panel **(M)** shows vascular Aβ deposits identified by counterstaining with Thioflavin-S. The scale bar is 50 μm in **(E, F)**, and **(G)**; 20 μm in **(A, C)**, and **(H–J)**; and 10 μm in **(B, D, K)**, and **(L)**.

Histopathological assessment of OXYS rats was performed at the age of 3, 7, 15–18, and 24 months. All Wistar rats at 3, 7, 15–18, and 24 months of age tested negative for Aβ deposits. In rare cases, we detected extracellular Aβ deposits in the brain of OXYS rats at 3–7 months of age with the antibodies used (Aβ_1–42_- and MOAB-2-specific antibodies), and Congo Red and Sirius Red. Aβ-immunoreactive deposits were readily detected in the brains of OXYS rats at 15–18 or 24 months of age. The affected areas included the cerebral cortex, hippocampal formation, thalamus, hypothalamus, and the brain stem, whereas the cerebellum was free of Aβ deposits. Compared to other areas, the cerebral cortex had the highest Aβ load (according to reactivity with both Aβ_1–42_- and MOAB-2-specific antibodies). Most of the amyloid was in the form of diffuse plaques that were positively immunoreactive with anti-Aβ antibodies (Fig. 1A, 1B, 1E, 1F, 1G, 1H, 1J, and 1L), Congo Red (Fig. 1C), and Sirius Red (Fig. 1D), but unreactive with the Thioflavin-S stain. Nevertheless, a small number of amyloid plaques did stain positively for Thioflavin-S; these plaques were defined as compact plaques that contain mainly fibrillar amyloid in the β-sheet-pleated conformation (Fig. 1I, 1J, and 1K). In addition, in OXYS rats aged 15–18 and 24 months, we saw vascular Aβ deposits (Fig. 1M).

We recently demonstrated that OXYS rats are characterized by overproduction of AβPP and of Aβ_1–42_ in the brain by the age of 12–13 months [7, 8]. APP is processed by β- and γ-secretases producing peptides of different length. Most of the Aβ produced by γ-secretase is the 40-mer form (Aβ_1–40_); however, the major Aβ species deposited in the plaques is the 42-mer variant (Aβ_1–42_), although this peptide represents only 5–10% of all Aβ produced. An increased Aβ_1–42_/Aβ_1–40_ ratio was proposed as a reliable indicator of neurochemical dementia diagnosis [9]. Accordingly, we measured the brain Aβ peptide levels by means of ELISA using human/rat Aβ_1–42_- and Aβ_1–40_-specific antibodies. The prefrontal cortex and hippocampus of 3, 12, and 24-month-old OXYS rats were analyzed separately because Aβ plaques developed at different rates in this region. We found that the levels of Aβ_1–42_ in the cortex and hippocampus were affected by age (F_2,42_ = 58.7, *p* = 0.001, and F_2,42_ = 48.6, *p* = 0.001, respectively) and genotype (F_1,42_ = 36.5, *p* = 0.001, and F_1,42_ = 45.5, *p* = 0.001, respectively). At the age of 12 and 24 months, OXYS rats had the highest level of Aβ_1–42_ in both the hippocampus and cortex compared to Wistar rats (*p* < 0.05; Table 1) but not at 3 months of age (*p* > 0.05). Note that at this age, OXYS rats show significant behavioral abnormalities and a cognitive decline [7, 8, 10, 11]. The levels of Aβ_1–40_ in the cortex and hippocampus were affected by age (F_2,42_ = 19.0, *p* = 0.001, and F_2,42_ = 85.1, *p* = 0.001, respectively) and genotype (only in the hippocampus: F_1,42_ = 4.5, *p* = 0.045). Indeed, the 3- and 12-month-old OXYS rats had a low level of Aβ_1–40_ in the hippocampus (*p* < 0.05), and there were no significant differences between the strains in the Aβ_1–40_ level in the cortex (Table 1).

**Table 1:** Levels of total Aβ_1–42_ and Aβ_1–40_ in the hippocampus and cortex of 3-, 12-, and 24-month-old OXYS and Wistar rats

Rat strain	Age, months	Hippocampus			Cortex		
		Aβ_1–40_ (pg/mg tissue)	Aβ_1–42_ (pg/mg tissue)	Aβ_1–42_/Aβ_1–40_ ratio	Aβ_1–40_ (pg/mg tissue)	Aβ_1–42_ (pg/mg tissue)	Aβ_1–42_/Aβ_1–40_ ratio
Wistar	3	32.4 ± 2.4	27.5 ± 3.4	0.8 ± 0.1	45.3 ± 2.7	30.7 ± 3.9	0.7 ± 0.1
	12	62.3 ± 2.8^#^	47.2 ± 6.9^#^	0.7 ± 0.1	69.5 ± 5.1^#^	56.6 ± 5.7^#^	0.8 ± 0.1
	24	68.9 ± 4.6	75.1 ± 12.1^#^	1.1 ± 0.3	65.4 ± 3.5	72.5 ± 3.6	1.1 ± 0.1
OXYS	3	25.1 ± 1.8^*^	32.9 ± 4.5	1.3 ± 0.2^*^	43.3 ± 1.1	33.6 ± 5.4	0.8 ± 0.1
	12	48.6 ± 4.5^*^^#^	79.6 ± 7.0^*^^#^	1.7 ± 0.1^*^	52.0 ± 7.2	103.5 ± 9.1^*^^#^	2.1 ± 0.1^*^^#^
	24	72.9 ± 4.0^#^	142.4 ± 10.0^*^^#^	2.0 ± 0.2^*^	77.6 ± 4.6^#^	176.9 ± 12.5^*^^#^	2.3 ± 0.3^*^

Aβ levels are presented as mean ± SEM (pg/mg brain wet tissue). The Aβ_1–42_/Aβ_1–40_ ratio is for total Aβ_1–42_ and total Aβ_1–40_ levels. Total Aβ is Aβ extracted in RIPA; *n* = 6 to 8 for all groups.

* Legend: statistically significant differences between the strains of the same age

# significant differences with the previous age within the strain.

Thus, our results were consistent with the higher plaque burden in the cortex according to the immunohistochemical analysis. The Aβ_1–42_/Aβ_1–40_ ratio increased greatly in both the hippocampus and cortex, with the cortex showing a stronger increase (Table 1). In rare cases, amyloid plaques were seen in 3- and 7-month-old OXYS rats. In the 15–18- and 24-month-old rats, the Aβ load measured by the immunohistochemical analysis with the Aβ-antibody was consistent with the Aβ levels measured using ELISA.

**Figure 2:**
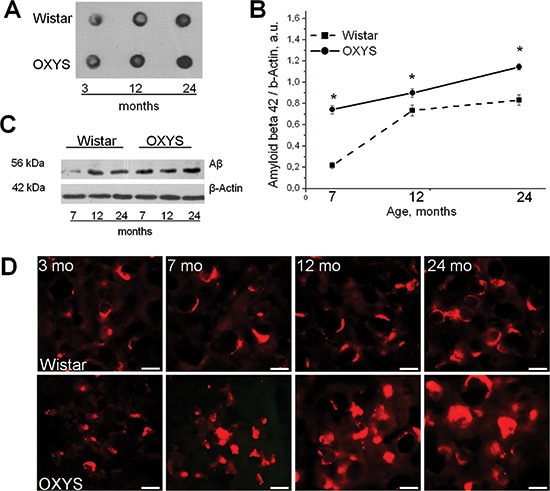
The pronounced accumulation of Aβ1–42 with age in the brain of OXYS rats **(A)** Dot-blot analysis of Aβ_1–42_ probed with the MOAB-2 antibody in the hippocampus of 3-, 12-, and 24-month-old OXYS and Wistar rats. Equal amounts of total protein were loaded on each dot. Aβ_1–42_ indeed accumulates more strongly by the age of 12 and 24 months in the brain of OXYS rats, but there were no differences in the MOAB-2 immunoreactivity between the two strains at 3 months of age. **(B–C)** Western blot analysis of TBS-soluble oligomeric Aβ_1–42_ probed with the specific antibody that detects Aβ_1–42_, and does not detect Aβ_1–40_, full-length APP, sAPP beta or sAPP alpha. The increased levels of Aβ_1–42_ detected according to quantitative in 7-, 12-, and 24-month-old OXYS rats compared to Wistar rats. Legend: *statistically significant differences between the strains of the same age. **(D)** Photomicrographs demonstrate the intensive aggregation of amyloid in the hippocampus of 7-, 12-, and 24- month-old OXYS rats but not at the age of 3 months, according to immunoreactivity with Aβ_1–42_-specific antibodies. The scale bar is 10 μm.

We next examined specificity of the Aβ_1–42_ accumulation by means of dot-blot analysis using MOAB-2, a monoclonal antibody that detects several conformational species of Aβ_1–42_, and does not detect APP. MOAB-2 is also selective for the more neurotoxic Aβ_1–42_ compared to Aβ_1–40_ [12]. The results showed that Aβ_1–42_ indeed accumulated more strongly with age in the brain of OXYS rats (Figure 2A).

To further assess the effects of Aβ pathology in OXYS rats, we analyzed the levels of soluble oligomeric Aβ_1–42_ fractions at the age of 7, 12, and 24 months using antibody that is reactive with Aβ_1–42_ and does not cross-react with Aβ_1–40_, full-length APP, sAPP beta or sAPP alpha. We found significantly increased levels of Aβ_1–42_ in 7-, 12-, and 24-month-old OXYS rats compared to Wistar rats, according to quantitative western blot analysis (F_1,36_ = 570.4, *p* = 0.0001; Figure 2B–2C) and with age in both rat strains (F_2,36_ = 456.9, *p* = 0.0001). In the 7-, 12-, and 24-month-old OXYS rats, the increased Aβ load in the hippocampus revealed by immunohistochemical analysis with Aβ_1–42_-specific antibodies (Figure 2D) was consistent with the Aβ levels measured by western blot analysis. Likewise, in the results observed in ELISA and dot-blot, statistically significant differences were apparent in Aβ levels between OXYS and Wistar rats at 12 and 24 months of age (Figure 2B). Moreover, our experiments seem to show for the first time that significant accumulation of Aβ_1–42_ in the brain of OXYS rats occurs by the age of 7 months.

### Increased expression of tau and the increased proportion of insoluble tau in OXYS rats

**Figure 3:**
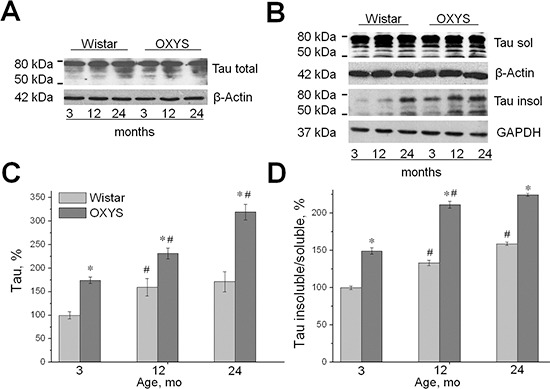
Increased expression of tau and the increased proportion of insoluble tau in OXYS rats. Expression of tau was analyzed by western blotting in the hippocampus of 3-, 12-, and 24-month-old OXYS and Wistar rats. The data are presented as a percentage of the data from 3-month-old Wistar rats in a group **(A, C)** The level of total tau (normalized to the level of β-actin) was significantly increased in OXYS rats compared to Wistar rats in each age group. **(B, D)** The proportion of insoluble tau and the ratio of sarkosyl-insoluble tau to sarkosyl-soluble tau (normalized to the level of GAPDH and β-actin, respectively) were significantly increased in the hippocampus of 3-, 12-, and 24-month-old OXYS rats compared to Wistar rats. The results were normalized to total protein and are presented as mean ± SEM of at least five independent experiments performed in duplicate. Legend: *statistically significant differences between the strains of the same age; ^#^significant differences with the previous age within the strain.

We used quantitative western blot analysis to determine the expression of tau in 3-, 12-, and 24-month-old OXYS and Wistar rats. We found that the tau protein content in the hippocampus of the two rat strains was affected by age (F_2,52_ = 7.3, *p* < 0.002) and genotype (F_1,52_ = 26.4, *p* < 0.0001). In line with our recent report [7], we found that in OXYS rats, the levels of tau in the hippocampus (estimated using a specific tau antibody) were significantly increased at 3, 12, and 24 months of age compared to Wistar rats (*p* < 0.05; Figure 3A, 3C). Content of the tau protein in the hippocampus of Wistar rats increased by 12 months of age (*p* < 0.05) and stayed at the same level until age 24 months, while in OXYS rats, these parameters increased strongly with age (*p* < 0.05).

To further evaluate the effects of the tau aberrations on accelerated brain aging in OXYS rats, we analyzed the levels of soluble and insoluble tau by sarkosyl fractionation in the two rat strains. The ratio of insoluble tau to soluble tau was calculated, and we found that the proportion of sarkosyl-insoluble tau (estimated with the Tau antibodies) was significantly increased in the hippocampus of 3-, 12-, and 24-month-old OXYS rats compared to Wistar rats (*p* < 0.05; Figure 3B, 3D). The proportion of sarkosyl-insoluble tau in the hippocampus of Wistar rats increased evenly with age (*p* < 0.05; Figure 3D), while in OXYS rats, these parameters increased strongly by age 12 months (*p* < 0.05) and stayed at the same level until age 24 months.

### Increased phosphorylation of sarkosyl-insoluble tau in OXYS rats

**Figure 4:**
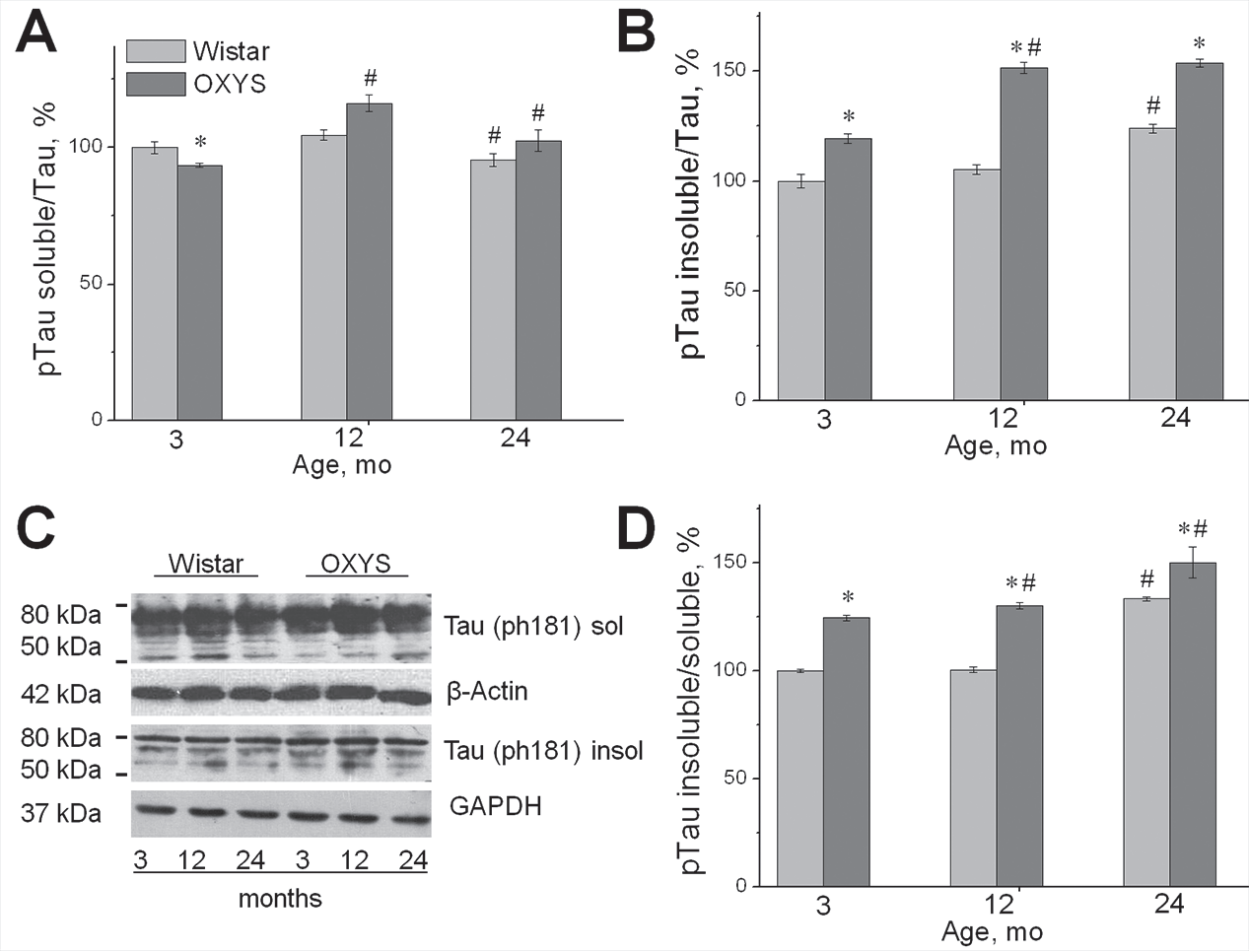
Increased phosphorylation of sarkosyl-insoluble tau in OXYS rats Expression of the sarkosyl-soluble fraction and sarkosyl-insoluble fraction recognized by the T181 antibodies was analyzed by western blotting in the hippocampus of 3-, 12-, and 24-month-old OXYS and Wistar rats. The data are presented as a percentage of the data from 3-month-old Wistar rats in a group. **(A, C)** The level of the sarkosyl-soluble fraction (normalized to β-actin) significantly decreased in OXYS rats only at 3 months of age compared to Wistar rats. **(B, C)** The level of the sarkosyl-insoluble fraction (normalized to GAPDH) significantly increased in the hippocampus of 3-, 12-, and 24-month-old OXYS rats compared to Wistar rats. **(D)** The proportion of insoluble phosphotau and the ratio of sarkosyl-insoluble phosphotau to sarkosyl-soluble phosphotau significantly increased in the hippocampus of 3-, 12-, and 24-month-old OXYS rats compared to the Wistar strain. The presented results were normalized to total protein and represent mean ± SEM of at least five independent experiments performed in duplicate. Legend: *statistically significant differences between the strains of the same age; ^#^significant differences compared to the previous age within the strain.

Phosphorylation levels of sarkosyl-soluble tau in OXYS and Wistar rats (detected by the T181 antibodies, normalized to tau detected by the Tau antibody; Figure 4A) were significantly decreased only in 3-month-old OXYS rats, compared to Wistar rats (*p* < 0.05; Figure 4A, 4C).

We then compared the phosphorylation levels of sarkosyl-insoluble tau (Figure 4B) between the strains. The levels of phosphotau recognized by the T181 antibody significantly increased in the sarkosyl-insoluble fraction from OXYS rats at the age of 3, 12, and 24 months compared to Wistar rat brains (*p* < 0.05; Figure 4B, 4C).

The ratio of insoluble phosphotau to soluble phosphotau was also calculated, and we found that the proportion of sarkosyl-insoluble phosphotau significantly increased in the hippocampus of 3-, 12-, and 24-month-old OXYS rats compared to Wistar rats (*p* < 0.05; Figure 4D). The proportion of sarkosyl-insoluble phosphotau in the hippocampus of OXYS rats increased with age (*p* < 0.05; Figure 4D), while in Wistar rats these parameters increased only by 24 months of age (*p* < 0.05).

### Mitochondrial degeneration in OXYS rats

**Figure 5:**
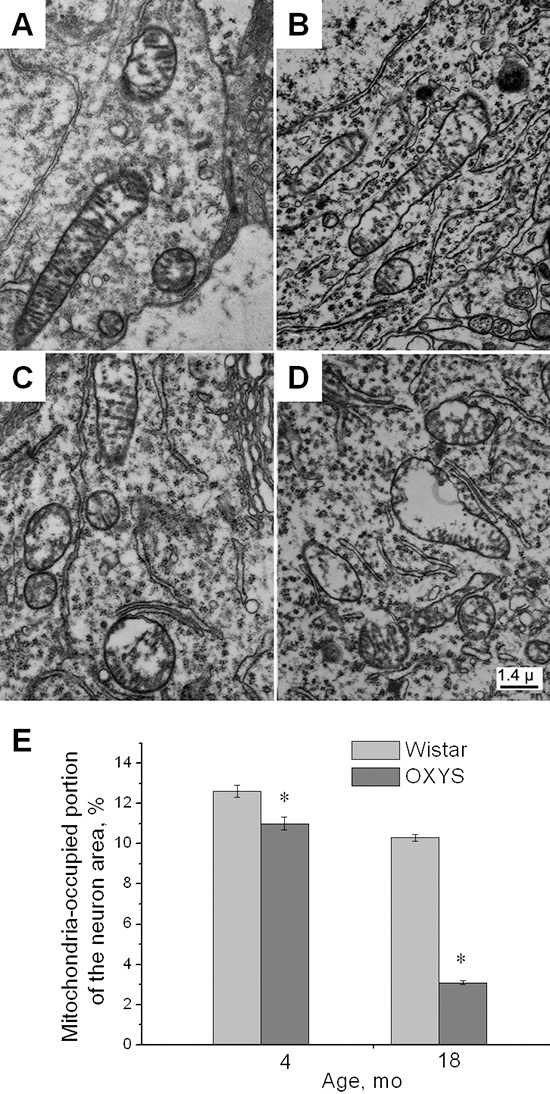
Alterations in the ultrastructure of the CA1 pyramidal neurons of OXYS rats **(A)** Mitochondria with normal ultrastructure in the cytoplasm of a neuron from a 4-month-old Wistar rat. **(B)** Mitochondria with a light matrix in the cytoplasm of a neuron of a 4-month-old OXYS rat. **(C)** Mitochondria with enlarged and empty intermembrane space in an 18-month-old Wistar rat. **(D)** Degradation of mitochondria in the neuronal cytoplasm of an 18-month-old OXYS rat. **(E)** The area occupied by mitochondria within the pyramidal neurons of the hippocampus as % of the total neuron area in the Wistar and OXYS rats. Legend: *statistically significant differences between the strains of the same age.

Increasing evidence points to a role of mitochondrial alterations upstream of Aβ and tau aberrations in AD [13]. Therefore, we examined ultrastructural characteristics of the mitochondrial apparatus in the pyramidal neurons of the CA1 region of the hippocampus of OXYS and Wistar rats at 4 and 18 months of age (Figure 5). Figure 5B shows large intramitochondrial cristae-free areas of low electron density in 4-month-old OXYS rats. In mitochondria retaining the more usual ultrastructure, the number of cristae was reduced, the intermembrane space was enlarged, and electron density of the matrix decreased (Figure 5B). Alterations in ultrastructure of the pyramidal neurons of OXYS rats became strongly pronounced by the age of 18 months. Figure 5D demonstrates dramatic degradation of the mitochondrial apparatus in 18-month-old OXYS rats. This apparatus appeared as a compressed electron-dense matrix with separate regions of tightly located membranes of the adjoining cristae as well as increased volume of the intermembrane space that corresponds to the de-energized state.

The results were quantified using stereotypic methods. For each of the four groups of OXYS and Wistar rats (4-month-old and 18-month-old groups) we calculated the mitochondria-occupied portion of the neuron area. Mathematical processing and statistical analysis of electron micrographs of the hippocampal neurons of OXYS and Wistar rats (Figure 5E) showed that even in young OXYS rats, mitochondria occupied as little as 11 ± 0.6% of the neuron area. This value decreased further to 3.1 ± 0.3% in 18-month-old OXYS rats. In Wistar rats, this parameter was 12.6 ± 0.5% at 4 months of age and 10.3 ± 0.3% at 18 months of age.

### Synapse losses and progressive neurodegeneration in OXYS rats

**Figure 6:**
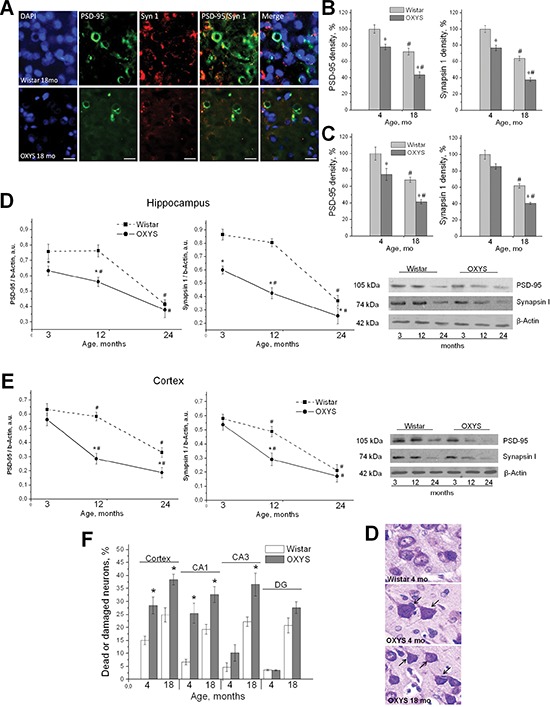
Synaptic losses and progressive neurodegeneration in OXYS rats **(A)** Immunohistochemical analysis using antibodies against PSD-95 (green) and synapsin I (Syn 1; red) on cortex neurons of 18-month-old OXYS and Wistar rats. The scale bar is 5 μm. **(B, C)** Quantification of the density of PSD-95 and synapsin I in the CA1 region **(B)** and in the cortex **(C)** of 4- and 18-month-old OXYS and Wistar rats. The data are presented as a percentage of the data from 4-month-old Wistar rats in a group. **(D, E)** Expression of PSD-95 and synapsin I was analyzed by western blotting in the hippocampus **(D)** and in the cortex **(E)** of 3-, 12-, and 24-month-old OXYS and Wistar rats. **(F)** Quantification of structural changes of hippocampal and cortical neurons of 4- and 18-month-old OXYS and Wistar rats (as a percentage of dead or damaged neurons). **(G)** Photomicrographs show the cortex neurons of 4-month-old Wistar rats and 4- and 18-month-old OXYS rats. The arrow shows damaged neurons in OXYS rats. The neurons were stained with Cresyl Violet. **(B–E)** Values are mean ± SEM. Legend: *statistically significant differences between the strains of the same age; ^#^significant differences with the previous age within the strain.

In AD, early hallmarks include the loss of synapses, and comparison of AD patients to age-matched control individuals shows that the density of synapses correlates strongly with cognitive impairment, suggesting that a loss of neuronal connections is associated with progression of the disease [14]. Therefore, to measure the density of synapses in OXYS rats, we used two synaptic markers in the hippocampus and prefrontal cortex of the animals: synapsin I, a marker of synaptic vesicles, and PSD-95, a postsynaptic marker (Figure 6A). We found that both pre- and postsynaptic density were significantly reduced with age in the hippocampus and cortex of both OXYS and Wistar rats (F_1,48_ = 8.7, *p* < 0.005, and F_1,48_ = 31.9, *p* < 0.0001 for the hippocampus, respectively, and F_1,48_ = 154.2, *p* < 0.0001, and F_1,54_ = 40.2, *p* < 0.0001 for cortex, respectively; Figure 6B, 6C). Nevertheless, 4- and 18-month-old OXYS rats showed reduced density of synapsin I and PSD-95 in the hippocampus compared to Wistar rats (F_1,48_ = 41.0, *p* < 0.0001, and F_1,48_ = 27.6, *p* < 0.0001, respectively; Figure 6B). The PSD-95 density in the cortex was significantly lower in 4- and 18-month-old OXYS rats compared to the Wistar strain (F_1,48_ = 25.7, *p* < 0.0001; Figure 6C). Synapsin I density was effected by genotype and was reduced in OXYS rats (F_1,48_ = 28.6, *p* < 0.0001; Figure 6C).

Next we assessed the integrity of presynaptic and postsynaptic elements by measuring the levels of synaptic proteins from the hippocampus and prefrontal cortex using western blot analysis. We saw an age-dependent reduction in synapsin I and PSD-95 expression in the hippocampus of both OXYS and Wistar rats (F_2,36_ = 334.2, *p* < 0.0001, and F_2,36_ = 227.1, *p* < 0.0001, respectively; Figure 6D), but these levels were significantly lower in 3-, 12-, and 24-month-old OXYS rats compared to Wistar rats (F_1,36_ = 338.0, *p* < 0.0001, and F_1,36_ = 93.7, *p* < 0.0001, respectively; Figure 6D). Synapsin I and PSD-95 expression levels in the prefrontal cortex decreased with age in both OXYS and Wistar rats too (F_2,36_ = 263.2, *p* < 0.0001, and F_2,36_ = 248.1, *p* < 0.0001, respectively; Figure 6E). The PSD-95 expression was significantly lower in 3-, 12-, and 24-month-old OXYS rats than in Wistar rats (F_1,36_ = 189.9, *p* < 0.0001; Figure 6E). Synapsin I expression was affected by genotype and was reduced in OXYS rats (F_1,36_ = 51.0, *p* < 0.0001; Figure 6E).

To estimate the neuronal loss, neuron counts in the prefrontal cortex and hippocampal subareas were performed on OXYS and Wistar rats at 4 and 18 months of age using stereological estimation of Cresyl Violet-labeled neuronal nuclei. Neurons were identified by their morphology. In OXYS rats, a significant neuronal loss was detected at 18 months of age in CA1, CA3, and dentate gyrus regions (*p* < 0.05) but not in the prefrontal cortex, compared to age-matched Wistar rats. We observed a 28% decrease in neuron density in CA1, a 47% decrease in CA3, and a 25% decrease in the dentate gyrus. Nonetheless, we observed significantly increased neuron density in all fields of the hippocampus and prefrontal cortex of OXYS rats at 4 months of age compared to age-matched Wistar rats (*p* < 0.05). We hypothesized that the enhanced neurogenesis at this age could serve as an endogenous compensatory mechanism of tissue repair. Quantification using stereological counts showed that significant structural changes of hippocampal and cortex neurons in OXYS rats developed at the age of 4 months (Figure 6F, 6G). The most pronounced neurodegenerative changes occurred in the CA1 region of the hippocampus and in the cortex of OXYS rats: there were 25% of dead or damaged neurons in CA1 and 28% of such neurons in the cortex (*p* < 0.05 for both; Figure 6F). With age, the neurodegeneration progressed, and at 18 months of age, the structural changes affected 32% of neurons of CA1 in the hippocampus of OXYS rats, 36% of neurons in CA3, 28% of neurons in the dentate gyrus, and 38% of neurons in the cortex (*p* < 0.05).

### Comparative SNP analyses in OXYS rats

Finally, we found it necessary to determine genetic variants that may be associated with the complex manifestation of AD-like pathology in OXYS rats. As described above, we performed cDNA sequencing of OXYS and WAG rats’ retina, with sequencing read depth of 40 million, by means of the RNA-Seq technology, Illumina. Control WAG rats had no significant signs of neurodegeneration, detectable on magnetic resonance imaging, and no behavioral alterations in the standard open-field test, according to our data (unpublished). Thus, the identified variants were supposed to be more specifically associated with the AD-like phenotype. It has been confirmed by numerous studies that the AD risk is affected by multiple genetic factors, and genetically, the disease is subdivided into familial cases and sporadic cases [15], although the majority of AD cases manifest themselves as a late-onset sporadic form. As expected [4], in the autosomal dominant early-onset form of AD, mutations in three genes were identified: *APP*, *PSEN1*, and *PSEN2*. These genes are responsible for approximately a half of all cases of the Mendelian form of the disease and have been included as causative genes in the new diagnostic guidelines for AD [16, 17]. In the more common late-onset AD form, the gene encoding *apolipoprotein E* (*APOE*), has been recognized as a major genetic risk factor [18]. Several promising disease risk genes for late-onset AD were identified in genome-wide association studies (GWAS): *PICALM*, *BIN1*, *CD33*, *CD2AP*, *CLU*, *CR1*, *EPHA1*, *ABCA7*, and the *MS4A* gene cluster [19–21]. These genes code for proteins that are central to the metabolism of cholesterol, activation of the immune system, and synaptic cell membrane processes. In the present study, the comparative SNP analysis was performed for coding regions of some of the above genes as well as some other genes associated with either late-onset or early-onset AD risk to some extent (35 genes in total; Table 2).

**Table 2:** Genes associated with Alzheimer's disease (AD) that were used in the comparative analysis of single nucleotide polymorphisms (SNPs)

Gene symbols	Brief information about some associations with AD	References
*APP*	Mutations lead to increased production of all β-amyloid proteins or β-amyloid 42. Mutations cause early-onset AD.	[16]
*PSEN1*	Mutations cause early-onset AD.	[17]
*PSEN2*		
*PICALM*	Putative functional variants (SNPs) in AD according to genome-wide association studies.	
*BIN1*		[20]
*CD33*		
*CD2AP*		[19]
*CLU*		
*CR1*		
*EPHA1*		
*ABCA7*		
*MS4A4A*		
*MS4A4E*		
*MS4A6E*		
*APOE*	Increased density of β-amyloid plagues and vascular deposits.	[57]
	The *APOEε4* allele frequency was found to be high in patients with late-onset AD.	[58]
	*APOE* is recognized as a major genetic risk factor in late-onset AD, although it is neither sufficient nor necessary to explain all occurrences of the disease.	[18]
*HADH2*		
*APBA1*		
*AGER*		
*GSK3B*		
*CDKHR1*		
*APPBP1*		
*APBA2*	The genes have the score according to physiological-genomic analysis in relation to AD.	[22]
*GAL*		
*APLP2*		
*CASP3*		
*SNCA*		
*SORL1*	SORL1 genetic variants are related to AD pathology.	[47]
	Recent reports from individual studies revealed significant associations with AD.	[48]
		[49]
	Involved in APP trafficking.	[50]
*NCSTN*	Components of the γ-secretase complex, which catalyzes the cleavage of amyloid precursor protein APP.	[51]
*PSENEN*		
*BACE1*	The aspartic protease BACE1 is the initiator enzyme for the formation of Aβ, a major constituent of amyloid plaques.	[52]
*FYN*	These were identified as candidate core regulatory mediators in differential coexpression correlation network analysis of the APOE4 and late-onset AD transcriptomic changes; these genes also encode known or novel modulators of late-onset AD associated amyloid β A4 precursor protein (APP) endocytosis and metabolism.	[53]
*SV2a*		
*RNF219*		
*TREM2*	*TREM2* mutation carriers with AD have more extensive brain atrophy than noncarriers with AD.	[54]
	Variants in the *TREM2* region are also associated with cerebrospinal fluid tau levels.	[55]
*PLD3*	Whole-exome sequencing in late-onset AD families was coupled with genotyping in large case-control series to identify *PLD3 V232M* as an AD risk factor.	[56]

The analysis was performed for coding regions using RNA-Seq data from the chorioretinal complex of OXYS and WAG rats.

Only three nonsynonymous SNPs located in two genes from the list—*Casp3* (1) and *Sorl1* (2)—were found in OXYS rats but not in WAG rats (Table 3). These three variants have not been described in Ensemble (http://www.ensembl.org). The consequence for all three SNPs was predicted as a missense mutation, but only one SNP, located in the *Sorl1* gene, was predicted to significantly affect protein structure (scored as “deleterious”) by the Variant Effect Predictor tool, VEP (SIFT algorithm). The rating for two other missense SNPs was “tolerated.”

**Table 3:** Nonsynonymous single nucleotide polymorphisms (SNPs) possibly associated with the nontransgenic Alzheimer's disease (AD) phenotype in rats

Gene symbol	Gene name	Location (chr:pos)	CDS position	Protein position	Codon change	AA change	SIFT (score)
*Casp3*	caspase 3	16:48561761	734	245	aCg/aTg	T/M	tolerable (0.22)
*Sorl1*	sortilin-related receptor, LDLR class A repeats-containing	8:44771636	3136	1046	Gtg/Ttg	V/L	tolerable (0.28)
*Sorl1*	sortilin-related receptor, LDLR class A repeats-containing	8:44786644	2230	744	Cgg/Tgg	R/W	deleterious (0.01)

SNPs in the selected genes (see the list in Table 2) were found in the comparative analysis of OXYS and WAG (control) rats’ cDNA sequence by means of RNA-Seq (Illumina). The SNP quality integral parameter threshold (in SAMtools/Bcftools) was Qual > 180, the consequence by the Variant Effect Predictor tool (www.ensembl.org) was *missense*. Only one SNP, located in the *Sorl1* gene, was predicted to significantly affect protein structure (scored as deleterious by the SIFT algorithm). Chr = chromosome; pos = position in base pairs; ref = reference; var = variant; AA = amino acid.

*Casp3*, which had the lowest physiogenomic score considering the results of physiological-genomic analysis in relation to AD [22], was examined in the present study for the association with generation of the pathologic Asp (421) truncation of tau in long-lasting fibrillary structures [23]. Only one SNP—located in exon 7 of the *Casp3* gene—was predicted as a missense mutation, but was scored as “tolerated” by the Variant Effect Predictor tool. Further studies are needed to determine the role of this variant in the development of AD-like pathology in OXYS rats.

The sorting protein-related receptor (sorLa) is involved in the control of Aβ peptide production and is less expressed in AD patients. Thus, sorLa most likely has an influence on the pathophysiology of AD, although the gene encoding sorLa (SORL1) has been evaluated as a susceptibility factor for late-onset AD with conflicting results. In the present study, we detected two missense mutations in the *Sorl1* gene in OXYS rats; one of these was predicted to significantly affect protein structure (“deleterious” according to VEP). It should be noted that the presence of SORL1 variants in late-onset and early-onset AD demonstrates that factors other than the mutation can impact the age of onset and penetrance of at least some variants associated with AD [24].

## DISCUSSION

We explored the role of Aβ in the pathogenesis of AD by analyzing the age-dependent profile of Aβ species in a nontransgenic rat model of AD. We report that OXYS rats show a significant age-dependent increase in the levels of soluble Aβ_1–40_ and Aβ_1–42_ and in extracellular Aβ deposits in several brain regions. The elevated Aβ_1–40_ and Aβ_1–42_ levels in the cortex of AD patients, even detectable in questionable dementia cases, strongly correlate with the subsequently observed cognitive decline [25]. It is believed that two factors are involved in the rate of Aβ deposition: the absolute level of Aβ_1–42_, and the Aβ_1–42_: Aβ_1–40_ ratio. OXYS rats that have simultaneously higher Aβ_1–42_ levels and Aβ_1–42_:Aβ_1–40_ ratios develop amyloid deposits after significant synaptic losses, neuronal cell death, mitochondrial structural abnormalities, and hyperphosphorylation of the tau protein. Based on the time course of Aβ accumulation, it is possible that this accumulation is second event of the degeneration process. Nevertheless, both phenomena could also run in parallel with different time courses. Our findings strongly support the idea that Aβ accumulation alone may not be sufficient to cause dementia in AD [2, 26]. Aβ is secreted to the extracellular milieu of the brain; this milieu is in contact with cerebrospinal fluid where Aβ is also detectable and performs important normal functions in the central nervous system, including neurogenesis, modulation of ion channel activity, kinase activation, synaptic plasticity, protection from oxidative damage, and enhancement of neuronal survival [27, 28, 29]. Impaired Aβ clearance, not increased synthesis, is believed to be the cause of the pathological accumulation of Aβ [5, 30].

AD is associated with dysfunction and eventually death of brain neurons [31]. Neuronal loss is usually prominent in the hippocampus and manifests itself further throughout the cerebral cortex, increasing with disease progression [32, 33].

Here we demonstrate a progressive loss of the neuronal populations of CA1 and CA3 and dentate gyrus regions of the hippocampus in OXYS rats at 18 months of age. We hypothesized that the phenomenon of enhanced neurogenesis in the hippocampus of OXYS rats at 4 months of age could serve as a compensatory mechanism of tissue repair or may be explained by the increased production of immature neurons. Jin and colleagues [34] have demonstrated increased hippocampal neurogenesis in patients with AD. They offer several possible reasons for the limited repair capacity of neurogenesis in AD: (i) the rate or extent of cell loss may be too high for quantitative replacement to be achieved; (ii) the neurons that are produced may be ineffective because they do not develop into fully mature functional neurons; (iii) the impairment of the microenvironment may have toxic effects on new neurons. It seems that these factors are involved in significant structural changes of neurons in 4-month-old OXYS rats, with an increase in the number of dead or damaged neurons by the age of 18 months. The dysfunctional neurogenesis in the hippocampus and cortex leads to subtle early manifestations of the disease, which could in turn render neurons more vulnerable to AD-like pathology and contribute to memory impairment [10, 11].

It is noteworthy that in 4-month-old OXYS rats, when we observed an increase in neuronal populations, there is already a significantly reduced level of synapsin I and PSD-95. A possible explanation of the observed synaptic losses could be persistently elevated neuronal activity, which can lead to excitotoxicity. With age in OXYS rats, the higher Aβ levels of secreted peptides may form neurotoxic fibrils that cause synaptic depression and eventually kill neurons. The finding that tau phosphorylation in the pathogenesis of AD is increased by amyloid toxicity raises the question about the possible cause of this process. On the other hand, our results demonstrate that hyperphosphorylation of the tau protein occurs before accumulation of Aβ. We found that already in 3-month-old OXYS rats, highly phosphorylated soluble tau is rapidly aggregated into the sarkosyl-insoluble toxic form, suggesting that hyperphosphorylation of tau may be an early impetus for the neural toxicity and synaptic losses that occur through transneuronal propagation of tau. Some authors hypothesized a sequence of progressive misfolding of tau proteins, circuit-based transfer to new cell populations, and deafferentation-induced degeneration as part of the tau-induced neurodegeneration [14]. Furthermore, the abnormal hyperphosphorylation of tau observed at 3, 12 and 24 months of age favors the idea that neurons that develop tau aberrations and deposition of Aβ may be lost, as we saw in 18-month-old OXYS rats.

Mitochondrial dysfunction and enhanced generation of reactive oxygen species are important features of AD [13, 35, 36]. The observation that oxidation of mitochondrial DNA occurs during aging and at the prodromal stage of AD strongly supports the notion that mitochondrial abnormalities are a causative factor of AD [37]. Analyzing our data, we can conclude that mitochondrial abnormalities play a key role in the accelerated brain aging of OXYS rats. The most pronounced ultrastructural changes of mitochondria are detected in OXYS rats already at the age of 4 months. Among these changes, there is cristae shrinkage resulting in formation of cristae-free regions inside mitochondria. In 18-month-old OXYS rats, mitochondrial reticulum is almost completely destroyed. The cause of the mitochondrial alterations in cells of AD patients is unknown but may involve aging and disease-related buildup of Aβ, oxidative stress, and reduced availability of cellular energy. Major functions of mitochondria in neurons include regulation of Ca^2+^ and redox signaling, developmental and synaptic plasticity, and arbitration of cell survival and death [13]. The age-associated cognitive decline in OXYS rats, particularly impairment of spatial learning [10–11], is associated with changes in the hippocampal synaptic plasticity including a deficit in long-term potentiation starting at 3–4 months of age [38]. Plasticity, the process through which synapses modulate their strength and neurons form new connections, performs a particularly important function in the response to injury and disease, including AD [27]. Our studies of oxidative stress markers in OXYS rats have demonstrated that accumulation of such markers as well as an imbalance in redox regulation appear after the main manifestations of accelerated senescence [39, 40]. Recently, we showed that OXYS rats exhibit significantly decreased expression for Cox8b (cytochrome c oxidase, subunit VIIIb) [41], an enzyme of the mitochondrial respiratory chain that is involved in the AD pathway [42]. Furthermore, at the age of 3 months, mRNA levels of many mitochondrial genes in the OXYS rats’ retina are the same as those of 18-month-old Wistar rats [41]. Accordingly, our data demonstrate that progressive mitochondrial dysfunction plays a major part in orchestrating accelerated brain aging and in the development of oxidative stress-associated disorders [43–45] in OXYS rats. These observations suggest that mild mitochondrial uncoupling is highly effective at supporting *in vivo* antioxidant mechanisms.

Even more intriguing is the question of which genes interact and how they work together to drive the progression of the disease or to augment the risk of AD. Here we performed comparative SNP analysis for coding regions of 35 genes that are associated with late-onset and early-onset AD risk. Among the gene variants that are found in OXYS rats but not in WAG rats, nonsynonymous SNPs are located only in the genes *Casp3* and *Sorl1*. Casp3 is associated with generation of a pathologic Asp (421) truncation of tau in long-lasting fibrillary structures [23]. The SORL1 gene has been studied as a susceptibility factor for late-onset AD, but the potentially pathogenic SORL1 mutations are also found in patients with early-onset AD [24]. In accordance with results of the present search for genetic variants, OXYS rats can be characterized as a nontransgenic model of AD without nonsynonymous AD mutations in the genes *App*, *Psen1*, and *Psen2*, which are identified in autosomal dominant early-onset AD. In summary, our results expand the spectrum of genotype-phenotype correlations for genes associated with AD, in a rat model. Further research is needed to determine whether our variants are indeed pathogenic.

In conclusion, on the basis of our results, we can theorize that multiple age-associated degenerative processes may precede the toxic accumulation of Aβ, which in turn triggers the final, currently irreversible stage of the sporadic form of AD and becomes a fatal hallmark event of the disease.

## MATERIALS AND METHODS

### Animals

Male senescence-accelerated OXYS rats (*n* = 124), age-matched male Wistar rats (*n* = 124), and 3-month-old WAG rats (*n* = 3) were obtained from the Breeding Experimental Animal Laboratory of the Institute of Cytology and Genetics, the Siberian Branch of the Russian Academy of Sciences (Novosibirsk, Russia). The OXYS rat strain was derived from the Wistar rat strain at the Institute of Cytology and Genetics as described earlier [10] and was registered in the Rat Genome Database (http://rgd.mcw.edu/). At this point, we have the 105th generation of OXYS rats with spontaneously developing cataract and accelerated senescence syndrome inherited in a linked manner.

At the age of four weeks, the pups were weaned, housed in groups of five animals per cage (57 × 36 × 20 cm), and kept under standard laboratory conditions (22 ± 2°C, 60% relative humidity, and 12 h light/12 h dark cycle). The animals were provided with standard rodent feed (PK-120-1, Laboratorsnab, Ltd., Russia) and water *ad libitum*. Animals between 3 and 24 months of age were used in the experiments, as described below. All animal care and experimental procedures were in compliance with European Directive-2010 of FELASA.

### Tissue preparation

All rats (*n* = 4 to 8 per genotype) at the age of 3, 4, 7, 12, 15–18, and 24 months were euthanized by CO_2_ asphyxiation; the brains were carefully removed. For histochemical and immunohistochemical assays, the right hemisphere was immediately fixed in 4% paraformaldehyde in phosphate-buffered saline (PBS) for 48 h followed by cryoprotection in 30% sucrose in PBS at 4°C for 2 days, and then the brains were frozen and stored at −70°C until further processing. For western blotting and ELISA, the hippocampus and prefrontal cortex were quickly separated from the left hemisphere, placed in microcentrifuge tubes for protein isolation, and frozen in liquid nitrogen. The tissues were stored at −70°C until further processing.

### Western blotting, dot-blot, and ELISA

Frozen tissues of the hippocampus and prefrontal cortex of OXYS and Wistar rats (*n* = 6 to 8 per genotype) at the age of 3, 12, and 24 months were homogenized in the protein lysis buffer RIPA (50 mM Tris-HCl pH 7.4, 150 mM NaCl, 1% Triton X-100, 1% sodium deoxycholate, 0.1% SDS, 1 mM EDTA) supplemented with a protease inhibitor cocktail (Sigma-Aldrich, USA). After incubation for 20 minutes on ice, the samples were centrifuged at 12,000 × *g* for 30 minutes at 4ºC, and the supernatants were transferred to new tubes. Total protein was quantified using the Bio-Rad Bradford Kit (Bio-Rad Laboratories, USA).

For western blotting analysis, approximately 30 μg of protein extracts from the hippocampus of the two rat strains was resolved on SDS-PAGE (8–12% gel) in the TGB running buffer (25 mM Tris base, 190 mM glycine, 0.1% SDS) and transferred to a nitrocellulose membrane (Amersham, USA). The membrane was blocked with 3% bovine serum albumin (BSA; Sigma-Aldrich) in Tris-buffered saline (TBS) with 0.1% Tween 20 (TBS-T) for 1 h at room temperature (RT), and incubated overnight at 4°C with antibody to tau (Abcam, USA), and for 1 h at RT with antibody to β-actin, synapsin I, or to PSD-95 (Abcam). Secondary antibodies were HRP-conjugated antibody (Abcam). After incubation with the respective secondary antibodies, chemiluminescence signals were measured and scanned, and intensity of the emission bands was quantified using the ImageJ software (NIH, Bethesda, MD). β-Actin was always used as an internal loading control.

To perform the dot-blot analysis, 10-μl aliquots of each protein sample from the hippocampus of the two rat strains were spotted on a nitrocellulose membrane (Amersham). Each membrane contained the entire set of samples. The membranes were allowed to dry completely at RT and were then blocked with 3% BSA (Sigma-Aldrich) in 0.1% TBS-T for 1 h. After that, the membranes were incubated for 1 h at RT with a mouse monoclonal antibody to MOAB-2 (Millipore, USA) and then for 1 h at RT with an HRP-conjugated antibody (Abcam). Next the chemiluminescence signals were measured and scanned, and the intensity of the emission bands was quantified using ImageJ (NIH, Bethesda, MD).

ELISA kits for Aβ_1–40_ and for Aβ_1–42_ (Wako, Japan) were performed for the hippocampus and cortex of the two rat strains according to the manufacturer's instructions. Quantitation was carried out based on measurement of optical density on a microtiter plate reader and recalculated as picograms of Aβ_1–40_ or Aβ_1–42_ protein per milligram of the hippocampus or cortex tissue.

For guantification of soluble Aβ_1–42_ each of the frozen tissue samples of the hippocampus of 7-, 12-, and 24-month-old OXYS rats (*n* = 6) and Wistar rats (*n* = 6) was homogenized on ice in 19 volumes of TBS (50 mM Tris-HCl pH 7.4, 150 mM NaCl) supplemented with the protease inhibitor cocktail (Sigma-Aldrich), followed by passing through a 27-gauge insulin needle (IMP, USA). The TBS-soluble fraction was collected by ultracentrifugation of the homogenate at 100,000 × *g* for 1 h at 4°C. When TBS-soluble fraction was run on SDS-PAGE (8% gels), transferred to nitrocellulose membranes, and probed with antibodies to Aβ_1–42_, β-actin (Abcam). β-Actin was used as an internal loading control. Secondary antibodies were an HRP-conjugated antibody (Abcam).

### Sarkosyl insolubility assay

The hippocampus of 3-, 12-, or 24-month-old OXYS rats (*n* = 6) or Wistar rats (*n* = 6) was homogenized in salt buffer (6 μl/mg; 100 mM MES pH 6.8, 750 mM NaCl, 1 mM EGTA, 0.5 mM MgSO_4_, 1 mM dithiothreitol, protease inhibitors). The homogenates were incubated for 20 min at 4°C and then centrifuged at 11,000 × *g* for 20 min at 4°C. The supernatants were then centrifuged at 100,000 × *g* for 60 min at 4°C. For isolation of insoluble proteins, the pellets were extracted twice with 1:10 (w/v) extraction buffer (10 mM Tris-HCl pH 7.4, 10% sucrose, 850 mM NaCl, 1 mM EGTA) and centrifuged at 15,000 × *g* for 20 min at 4°C. The supernatants were combined and sarkosyl was added to a final concentration of 1%, followed by 1 h of incubation at RT on a rotating (inverting) stirrer. The samples were then centrifuged at 100,000 × g for 45 min at 4°C. The supernatants were then collected (sarkosyl-soluble fraction). The pellets were resuspended in 0.4 μl/mg 50 mM Tris-HCl (pH 7.4) and centrifuged at 15,000 × *g* for 20 min at 4°C. The supernatant was then collected (sarkosyl-insoluble fraction). Sarkosyl-insoluble and -soluble fractions were run on SDS-PAGE (8% gels), transferred to nitrocellulose membranes, and probed with antibodies to the tau protein, phosphoT181, β-actin (Abcam), or GAPDH (Santa Cruz, USA). β-Actin and GAPDH were always used as internal loading controls. Secondary antibodies were an HRP-conjugated (Abcam).

### Histochemistry and immunohistochemistry

Brain sagittal sections (20 μm thick) of 3-, 4-, 7-, 12-, 15–18-, and 24-month-old OXYS rats (*n* = 4 to 6) and Wistar rats (*n* = 4) were prepared using the Microm HM-505 N cryostat (Microm, Germany) at −20°C and transferred onto Polysine-glass slides (Menzel-Glaser, Braunschweig, Germany). For immunohistochemical analysis, the slices were transferred to TBS and washed. Then, to inhibit the endogenous peroxidase activity, the slices were incubated in a 0.3% H_2_O_2_ solution in TBS for 15 min. After serial washes in TBS, the slices were incubated for 15 min in TBS-plus (with 0.1% Triton X-100) and in 3% BSA (Sigma-Aldrich) in TBS for 1 h at RT to block nonspecific binding sites and to permeabilize the tissues, and were then incubated overnight with primary antibodies at 4°C. Primary antibodies were all diluted in 1% BSA in TBS to MOAB-2 (Millipore) and Aβ_1–42_ (Abcam). After serial washing in TBS, the slices were incubated with HRP secondary antibodies (Abcam) for 1 h at RT and washed with TBS. Then Steady DAB/Plus Buffer (Abcam) was used as a chromogen. The slices were washed in PBS and coverslipped with CC/Mount (Sigma-Aldrich). The different brain regions were defined according to the atlas developed by [46].

For an immunofluorescence assay, after serial washes with TBS, the slices were incubated for 15 min in TBS-plus (with 0.1% Triton X-100) and in 3% BSA (Sigma-Aldrich) in TBS for 1 hr at RT to block nonspecific binding sites and to permeabilize the tissues, and then were incubated overnight with primary antibodies at 4°C. Primary antibodies were all diluted in 1% BSA in TBS: antibodies to Aβ_1–42_, synapsin I, PSD-95 (Abcam) and MOAB-2 (Millipore). After serial washing with TBS, the slices were incubated with secondary antibodies: antibody conjugated to Texas Red (Abnova, USA), antibodies conjugated to DyLight-650, FITC, Alexa Fluor 488 (Abcam) for 1 h at RT and were then washed in TBS. The slices were washed in PBS and coverslipped with the mounting medium with DAPI (Abcam). Negative controls were processed in an identical manner except that a primary antibody was not included.

To visualize plaque morphology, Congo Red and Cresyl Violet or Sirius Red (Sigma-Aldrich) and Cresyl Violet double staining was performed according to standard protocols.

For Thioflavin-S staining, brain slices were mounted on slides and washed with PBS for 10 min. After the PBS wash, the slices were incubated in 1% Thioflavin-S (Sigma-Aldrich) for 8 min. After washing out excess Thioflavin-S with PBS, the slices were coverslipped with CC/Mount (Sigma-Aldrich). Images were captured using a fluorescence microscope.

### Estimation of synapse density

We captured images of the same area of the middle molecular layer of the prefrontal cortex and the CA1 pyramidal layer of the hippocampus of 4- and 18-month-old OXYS an Wistar rats (*n* = 3 to 5 animals per group) on every 4–6 serial slices per animal using an Axioplan 2 imaging microscope (Zeiss; Germany). Six frames 24.4 × 28.8 μm with the total area of 4216.3 μm^2^ were selected for counting of synapse density per section. In these images, we counted clusters stained with anti-PSD-95 and anti-synapsin I antibodies. We then determined the density of these clusters per 100 μm^2^ in each image and performed statistical analysis of these results. The various brain regions were defined according to the atlas developed by [46]. Standard immunofluorescence techniques were used to stain synapsin I and PSD-95 (Abcam) as described above. The data were analyzed using ImageJ (NIH, Bethesda, MD).

### Estimation of neuron numbers and morphology of neurons

Quantification of the neuron numbers in the prefrontal cortex and hippocampus was conducted in the right hemisphere of 4- and 18-month-old OXYS and Wistar rats (*n* = 5 animals per group). Mounted sections were incubated at 60°C in a Cresyl Violet solution for 3 minutes, briefly washed in distilled H_2_O and dehydrated in 70%, 95%, and 100% ethanol. All slices were immersed in xylene for 2–5 min and coverslipped with CC/Mount (Sigma-Aldrich). For the estimates of neuron numbers in the middle molecular layer of the prefrontal cortex, dentate gyrus granule cell layer, and CA1 and CA3 pyramidal layer, a set of every 4–6 serial sections per animal were used. A 100× objective (Axioplan 2, Zeiss, Germany) was used to count >200 neurons per visual field. The different brain regions were defined according to the atlas developed by [43]. The dead and damaged neurons were identified by their morphology. Images of the same area of brain regions were analyzed using ImageJ (NIH, Bethesda, MD). The data in Figure 6 were presented as a percentage of the dead and damaged neurons among all neurons in each visual field of a brain region of a group.

### Electron microscopy and calculations

For electron microscopic examination, small samples from the hippocampus (2 × 2 × 2 mm) were taken from the brain of 4- and 18-month-old OXYS and Wistar rats (*n* = 5 animals per group). The samples were fixed with a 2.5% glutaraldehyde solution in 0.1 M sodium cacodylate buffer (pH 7.2) for 1 h, rinsed with 0.1 sodium cacodylate buffer, and postfixed in 1% osmium tetroxide in the same buffer for 1 h. After that, the samples were washed with water and placed in a 1% uranyl acetate aqueous solution in the dark at RT for 1 h. The samples were then dehydrated using a graded series of ethanol and acetone and embedded in Agar 100 resin. Ultrathin slices were double-stained with uranyl acetate and lead citrate and examined under a transmission electron microscope (JEM 100 SX, JEOL) at the Interinstitutional Shared Centre for Microscopic Analysis of Biological Objects, the Institute of Cytology and Genetics, SB RAS.

For quantitative analysis, electron-transparent regions were identified on electron micrographs of pyramidal neurons of the CA1 region (50 photos for each group of animals). Then all mitochondria located in these regions were painted. The photos were processed in Adobe Photoshop; for each photo, the following parameter was determined: the total area of mitochondria located in the electron-transparent neuron areas. Then the mitochondria-occupied portion of the neuron area was calculated.

### RNA isolation

Three-month-old OXYS rats (*n* = 3) and WAG (control) rats (*n* = 3) were euthanized using CO_2_ inhalation. The chorioretinal complex was excised rapidly, placed in RNAlater (Ambion), frozen, and stored at −70°C prior to analysis. Frozen rat tissues were lysed using the TRIzol Reagent (Invitrogen), and total RNA was isolated according to the manufacturer's protocol. RNA quality and quantity were assessed using Agilent Bioanalyser (Agilent).

### Illumina sequencing

Over 40 million single-end reads of 50-bp length were obtained for each sample of OXYS (in triplicate) and WAG (1 pooled sample from 3 animals) male retinal RNAs, using Illumina nonstranded sequencing (on an Illumina GA IIx at the “Genoanalitika” Lab) in accordance with standard Illumina protocols (mRNA-Seq Sample Prep Kit). Briefly, polyA-tailed mRNA was purified from total RNA using Sera-Mag Magnetic Oligo (dT) beads and then fragmented into small pieces using divalent cations and heating. Using reverse transcriptase and random primers, the cleaved RNA fragments were then used to synthesize the first- and second-strand cDNAs. The cDNA was processed in an end repair reaction with T4 DNA polymerase and Klenow DNA polymerase in order to blunt the termini. An A base was then added to the 3′ end of the blunted phosphorylated DNA fragments, and an Illumina adaptor with a single T base overhang at its 3′ end was then ligated to the end of the DNA fragment, for hybridization in a single-read flow cell. After the ligation reaction, a size range of cDNA templates was selected, and these fragments were amplified on a cluster station using Single-Read Cluster Generation Kit v2. Sequencing-by-synthesis (SBS) of 50-nucleotide length was carried out on a Genome Analyzer IIx running the SCS2.8 software using SBS v4 reagents (Illumina).

### Mapping and SNP discovery

After barcode trimming, the sequencing data were tested for quality using the FastQC software and mapped to the *Rattus norvegicus* reference genome assembly RGSC 5.0 (Ensemble release 75) using Bowtie 2 or TopHat v2.0.4. The SNP positions within the aligned reads relative to the reference genome were identified using the pileup function in SAMtools (v. 0.1.17) utilities [44, http://samtools.sourceforge.net/index.shtml]. Using the various filter commands, SNPs were predicted for various positions with a minimum mapping quality (Q) of 100. These parameters ensure high-quality, reliable mapping of the reads, which is important for variant calling. Using custom-designed Perl scripts, the VCF files were converted into MySQL tables. The further analysis was based on the comparison of SNPs specific for OXYS and WAG rats using MySQL 5.0 queries (Oracle Corporation).

### Prediction of the SNP phenotypes

The SNPs that were present in OXYS rats but absent in control WAG rats were considered possibly associated with the nontransgenic AD phenotype. The effect of a variant amino acid substitution on protein function was predicted using the Variant Effect Predictor web tool [http://www.ensembl.org/]; the consequence type, SIFT score, and prediction were obtained for each variant. Generally, SIFT scores of 0–0.05 were classified as “deleterious” and 0.05–1.00 as “tolerated.”

### Statistics

Either the Mann–Whitney *U* test or analysis of variance (ANOVA) was used to determine whether the differences existed between experimental mean values. The *p* values < 0.05 were assumed to denote statistical significance. All statistical calculations were performed using the software package Statistica 6.0 (USA). The data were presented as mean ± SEM.

## References

[1] Morley JE, Armbrecht HJ, Farr SA, Kumar VB (2012). The senescence accelerated mouse (SAMP8) as a model for oxidative stress and Alzheimer's disease. Biochim Biophys Acta.

[2] Drachman DA (2014). The amyloid hypothesis, time to move on: Amyloid is the downstream result, not cause, of Alzheimer's disease. Alzheimers Dement.

[3] Morley JE, Farr SA (2014). The role of amyloid-beta in the regulation of memory. Biochem Pharmacol.

[4] Krstic D, Knuesel I (2013). Deciphering the mechanism underlying late-onset Alzheimer disease. Nat Rev Neurol.

[5] Castellano JM, Deane R, Gottesdiener AJ, Verghese PB, Stewart FR, West T, Paoletti AC, Kasper TR, DeMattos RB, Zlokovic BV, Holtzman DM (2012). Low-density lipoprotein receptor overexpression enhances the rate of brain-to-blood Aβ clearance in a mouse model of β-amyloidosis. Proc Natl Acad Sci U S A.

[6] Kozhevnikova OS, Korbolina EE, Stefanova NA, Muraleva NA, Orlov YL, Kolosova NG (2013). Association of AMD-like retinopathy development with an Alzheimer's disease metabolic pathway in OXYS rats. Biogerontology.

[7] Stefanova NA, Muraleva NA, Skulachev VP, Kolosova NG (2014). Alzheimer's disease-like pathology in senescence-accelerated OXYS rats can be partially retarded with mitochondria-targeted antioxidant SkQ1. J Alzheimers Dis.

[8] Stefanova NA, Kozhevnikova OS, Vitovtov AO, Maksimova KY, Logvinov SV, Rudnitskaya EA, Korbolina EE, Muraleva NA, Kolosova NG (2014). Senescence-accelerated OXYS rats: a model of age-related cognitive decline with relevance to abnormalities in Alzheimer disease. Cell Cycle.

[9] Wolfe MS (2007). When loss is gain: reduced presenilin proteolytic function leads to increased Abeta42/Abeta40. Talking Point on the role of presenilin mutations in Alzheimer disease EMBO.

[10] Stefanova NA, Fursova A, Kolosova NG (2010). Behavioral effects induced by mitochondria-targeted antioxidant SkQ1 in Wistar and senescence-accelerated OXYS rats. J Alzheimers Dis.

[11] Stefanova NA, Fursova A, Sarsenbaev KN, Kolosova NG (2011). Effects of Cistanche deserticola on behavior and signs of cataract and retinopathy in senescence-accelerated OXYS rats. J Ethnopharmacol.

[12] Youmans KL, Tai LM, Kanekiyo T, Stine WB, Michon SC, Nwabuisi-Heath E, Manelli AM, Fu Y, Riordan S, Eimer WA, Binder L, Bu G, Yu C, Hartley DM, LaDu MJ (2012). Intraneuronal Aβ detection in 5xFAD mice by a new Aβ-specific antibody. Mol Neurodegener.

[13] Mattson MP, Gleichmann M, Cheng A (2008). Mitochondria in neuroplasticity and neurological disorders. Neuron.

[14] de Calignon A, Polydoro M, Suárez-Calvet M, William C, Adamowicz DH, Kopeikina KJ, Pitstick R, Sahara N, Ashe KH, Carlson GA, Spires-Jones TL, Hyman BT (2012). Propagation of tau pathology in a model of early Alzheimer's disease. Neuron.

[15] Piaceri I, Nacmias B, Sorbi S (2013). Genetics of familial and sporadic Alzheimer's disease. Front Biosci (Elite Ed).

[16] Jack CR, Albert MS, Knopman DS, McKhann GM, Sperling RA, Carrillo MC, Thies B, Phelps CH (2011). Introduction to the recommendations from the National Institute on Aging-Alzheimer's Association workgroups on diagnostic guidelines for Alzheimer's disease. Alzheimers Dement.

[17] Yagi R, Miyamoto R, Morino H, Izumi Y, Kuramochi M, Kurashige T, Maruyama H, Mizuno N, Kurihara H, Kawakami H (2014). Detecting gene mutations in Japanese Alzheimer's patients by semiconductor sequencing. Neurobiol Aging.

[18] Schmidt V, Carlo AS, Willnow TE (2014). Apolipoprotein E receptor pathways in Alzheimer disease. Wiley Interdiscip Rev Syst Biol Med.

[19] Kamboh MI, Demirci FY, Wang X, Minster RL, Carrasquillo MM, Pankratz VS, Younkin SG, Saykin AJ, Jun G, Baldwin C, Logue MW, Buros J, Farrer L (2012). Genome-wide association study of Alzheimer's disease. Transl Psychiatry.

[20] Shi H, Belbin O, Medway C, Brown K, Kalsheker N, Carrasquillo M, Proitsi P, Powell J, Lovestone S, Goate A, Younkin S, Passmore P (2012). Genetic and Environmental Risk for Alzheimer's Disease Consortium, Morgan K; Alzheimer's Research UK Consortium. Genetic variants influencing human aging from late-onset Alzheimer's disease (LOAD) genome-wide association studies (GWAS). Neurobiol Aging.

[21] Tan LI, Yu JT, Zhang W, Wu ZC, Zhang Q, Liu QY, Wang W, Wang HF, Ma XY, Cui WZ (2013). Association of GWAS-linked loci with late-onset Alzheimer's disease in a northern Han Chinese population. Alzheimers Dement.

[22] Wiwanitkit V (2013). Physiological genomics analysis for Alzheimer's disease. Ann Indian Acad Neurol.

[23] Jarero-Basulto JJ, Luna-Muñoz J, Mena R, Kristofikova Z, Ripova D, Perry G, Binder LI, Garcia-Sierra F (2013). Proteolytic cleavage of polymeric tau protein by caspase-3: implications for Alzheimer disease. J Neuropathol Exp Neurol.

[24] Yin RH, Yu JT, Tan L (2014). The Role of SORL1 in Alzheimer's Disease. Mol Neurobiol.

[25] Näslund J, Haroutunian V, Mohs R, Davis KL, Davies P, Greengard P, Buxbaum JD (2000). Correlation between elevated levels of amyloid beta-peptide in the brain and cognitive decline. JAMA.

[26] Struble RG, Ala T, Patrylo PR, Brewer GJ, Yan XX (2010). Is brain amyloid production a cause or a result of dementia of the Alzheimer's type?. J Alzheimers Dis.

[27] Parihar MS, Brewer GJ (2010). Amyloid-beta as a Modulator of Synaptic Plasticity. J Alzheimers Dis.

[28] Stanga S, Lanni C, Govoni S, Uberti D, D'Orazi G, Racchi M (2010). Unfolded p53 in the pathogenesis of Alzheimer's disease: is HIPK2 the link?. Aging.

[29] Kumar S, Walter J (2011). Phosphorylation of amyloid beta (Aβ) peptides - a trigger for formation of toxic aggregates in Alzheimer's disease. Aging (Albany NY).

[30] Mawuenyega KG, Sigurdson W, Ovod V, Munsell L, Kasten T, Morris JC, Yarasheski KE, Bateman RJ (2010). Decreased Clearance of CNS beta-Amyloid in Alzheimer's Disease. Science.

[31] Song B, Davis K, Liu XS, Lee HG, Smith M, Liu X (2011). Inhibition of Polo-like kinase 1 reduces beta-amyloid-induced neuronal cell death in Alzheimer's disease. Aging (Albany NY).

[32] Brun A, Englund E (1981). Regional pattern of degeneration in Alzheimer's disease: neuronal loss and histopathological grading. Histopathology.

[33] Wright AL, Zinn R, Hohensinn B, Konen LM, Beynon SB, Tan RP, Clark IA, Abdipranoto A, Vissel B (2013). Neuroinflammation and Neuronal Loss Precede Ab Plaque Deposition in the hAPP-J20 Mouse Model of Alzheimer's Disease. PloS ONE.

[34] Jin KL, Peel AL, Mao XO, Xie L, Cottrell BA, Henshall DC, Greenberg DA (2004). Increased hippocampal neurogenesis in Alzheimer's disease. Proc Natl Acad Sci U S A.

[35] Dumont M, Lin MT, Beal MF (2010). Mitochondria and antioxidant targeted therapeutic strategies for Alzheimer's disease. J Alzheimers Dis.

[36] Jaszberenyi M, Rick FG, Szalontay L, Block NL, Zarandi M, Cai RZ, Schally AV (2012). Beneficial effects of novel antagonists of GHRH in different models of Alzheimer's disease. Aging (Albany NY).

[37] Santos RX, Correia SC, Zhu X, Smith MA, Moreira PI, Castellani RJ, Nunomura A, Perry G (2013). Mitochondrial DNA oxidative damage and repair in aging and Alzheimer's disease. Antioxid Redox Signal.

[38] Beregovoy NA, Sorokina NS, Starostina MV, Kolosova NG (2011). Age-specific peculiarities of formation of long-term posttetanic potentiation in OXYS rats. Bull Exp Biol Med.

[39] Kolosova NG, Grishanova A, Krysanova ZhS, Zueva TV, Sidorova IuA, Sinitsyna OI (2004). Age-related changes in protein and lipid oxidation in the liver of prematurely aging rats OXYS. Biomed Khim.

[40] Kolosova NG, Shcheglova TV, Sergeeva SV, Loskutova LV (2006). Long-term antioxidant supplementation attenuates oxidative stress markers and cognitive deficits in senescent-accelerated OXYS rats. Neurobiol Aging.

[41] Kozhevnikova OS, Korbolina EE, Ershov NI, Kolosova NG (2013). Rat retinal transcriptome: effects of aging and AMD-like retinopathy. Cell Cycle.

[42] Maurer I, Zierz S, Moller HJ (2000). A selective defect of cytochrome c oxidase is present in brain of Alzheimer disease patients. Neurobiol Aging.

[43] Shabalina IG, Kolosova NG, Grishanova A, Solov'ev VN, Salganik RI, Solov'eva NA (1995). Oxidative phosphorylation activity, F0F1-ATPase and level of liver mitochondrial cytochromes in rats with congenitally increased ability for free radical formation. Biokhimiia.

[44] Kolosova NG, Aidagulova SV, Nepomnyashchikh GI, Shabalina IG, Shalbueva NI (2001). Dynamics of structural and functional changes in hepatocyte mitochondria of senescence-accelerated OXYS rats. Bull Exp Biol Med.

[45] Vays VB, Eldarov CM, Vangely IM, Kolosova NG, Bakeeva LE, Skulachev VP (2014). Antioxidant SkQ1 delays sarcopenia-associated damage of mitochondrial ultrastructure. Aging (Albany NY).

[46] Paxinos G, Watson C (2007). The Rat Brain in Stereotaxic Coordinates.

[47] Alexopoulos P, Guo LH, Kratzer M, Westerteicher C, Kurz A, Perneczky R (2011). Impact of SORL1 single nucleotide polymorphisms on Alzheimer's disease cerebrospinal fluid markers. Dement Geriatr Cogn Disord.

[48] Elias-Sonnenschein LS, Bertram L, Visser PJ (2012). Relationship between genetic risk factors and markers for Alzheimer's disease pathology. Biomark Med.

[49] Willnow TE, Andersen OM (2013). Sorting receptor SORLA- a trafficking path to avoid Alzheimer disease. J Cell Sci.

[50] Capsoni S, Carlo AS, Vignone D, Amato G, Criscuolo C, Willnow TE, Cattaneo A (2013). SorLA deficiency dissects amyloid pathology from tau and cholinergic neurodegeneration in a mouse model of Alzheimer's disease. J Alzheimers Dis.

[51] Hansson CA, Frykman S, Farmery MR, Tjernberg LO, Nilsberth C, Pursglove SE, Ito A, Winblad B, Cowburn RF, Thyberg J, Ankarcrona M (2004). Nicastrin, presenilin, APH-1, and PEN-2 form active gamma- secretase complexes in mitochondria. J Biol Chem.

[52] Kandalepas PC, Vassar R (2014). The Normal and Pathologic Roles of the Alzheimer's β-secretase, BACE1. Curr Alzheimer Res.

[53] Rhinn H, Fujita R, Qiang L, Cheng R, Lee JH, Abeliovich A (2013). Integrative genomics identifies APOE ε4 effectors in Alzheimer's disease. Nature.

[54] Rajagopalan P, Hibar DP, Thompson PM (2013). TREM2 and neurodegenerative disease. N Engl J Med.

[55] Cruchaga C, Kauwe JS, Harari O, Jin SC, Cai Y, Karch CM, Benitez BA, Jeng AT, Skorupa T, Carrell D, Bertelsen S, Bailey M (2013). GWAS of cerebrospinal fluid tau levels identifies risk variants for Alzheimer's disease. Neuron.

[56] Cruchaga C, Karch CM, Jin SC, Benitez BA, Cai Y, Guerreiro R, Harari O, Norton J, Budde J, Bertelsen S, Jeng AT, Cooper B, Skorupa T (2014). Rare coding variants in the phospholipase D3 gene confer risk for Alzheimer's disease. Nature.

[57] Saunders AM, Strittmatter WJ, Schmechel D, George-Hyslop PH, Pericak-Vance MA, Joo SH, Rosi BL, Gusella JF, Crapper-MacLachlan DR, Alberts MJ (1993). Association of apolipoprotein E allele epsilon 4 with late-onset familial and sporadic Alzheimer's disease. Neurology.

[58] Genin E, Hannequin D, Wallon D, Sleegers K, Hiltunen M, Combarros O, Bullido MJ, Engelborghs S, De Deyn P, Berr C, Pasquier F, Dubois B, Tognoni G (2011). APOE and Alzheimer disease: a major gene with semi-dominant inheritance. Mol Psychiatry.

